# Context dependent regulatory patterns of the androgen receptor and androgen receptor target genes

**DOI:** 10.1186/s12885-016-2453-4

**Published:** 2016-07-04

**Authors:** Jan Roger Olsen, Waqas Azeem, Margrete Reime Hellem, Kristo Marvyin, Yaping Hua, Yi Qu, Lisha Li, Biaoyang Lin, XI- Song Ke, Anne Margrete Øyan, Karl- Henning Kalland

**Affiliations:** Department of Clinical Science, University of Bergen, Bergen, Norway; Centre for Cancer Biomarkers, University of Bergen, Bergen, Norway; Department of Microbiology, Haukeland University Hospital, Bergen, Norway; Cancer Institute, Second Affiliated Hospital, College of Medicine, Zhejiang University, Hangzhou, People’s Republic of China; Department of Urology, University of Washington, Seattle, WA USA; Laboratory Bld. 5. etg, Bergen Health, Bergen, NO-5021 Norway

**Keywords:** Human prostate cancer, Androgen receptor, Differentiation, Epithelial to mesenchymal transition, Stem cell

## Abstract

**Background:**

Expression of the androgen receptor (AR) is associated with androgen-dependent proliferation arrest and terminal differentiation of normal prostate epithelial cells. Additionally, activation of the AR is required for survival of benign luminal epithelial cells and primary cancer cells, thus androgen deprivation therapy (ADT) leads to apoptosis in both benign and cancerous tissue. Escape from ADT is known as castration-resistant prostate cancer (CRPC). In the course of CRPC development the AR typically switches from being a cell-intrinsic inhibitor of normal prostate epithelial cell proliferation to becoming an oncogene that is critical for prostate cancer cell proliferation. A clearer understanding of the context dependent activation of the AR and its target genes is therefore desirable.

**Methods:**

Immortalized human prostate basal epithelial EP156T cells and progeny cells that underwent epithelial to mesenchymal transition (EMT), primary prostate epithelial cells (PrECs) and prostate cancer cell lines LNCaP, VCaP and 22Rv1 were used to examine context dependent restriction and activation of the AR and classical target genes, such as KLK3. Genome-wide gene expression analyses and single cell protein analyses were applied to study the effect of different contexts.

**Results:**

A variety of growth conditions were tested and found unable to activate AR expression and transcription of classical androgen-dependent AR target genes, such as *KLK3*, in prostate epithelial cells with basal cell features or in mesenchymal type prostate cells. The restriction of androgen- and AR-dependent transcription of classical target genes in prostate basal epithelial cells was at the level of AR expression. Exogenous AR expression was sufficient for androgen-dependent transcription of AR target genes in prostate basal epithelial cells, but did not exert a positive feedback on endogenous AR expression. Treatment of basal prostate epithelial cells with inhibitors of epigenetic gene silencing was not efficient in inducing androgen-dependent transcription of AR target genes, suggesting the importance of missing cofactor(s).

**Conclusions:**

Regulatory mechanisms of AR and androgen-dependent AR target gene transcription are insufficiently understood and may be critical for prostate cancer initiation, progression and escape from standard therapy. The present model is useful for the study of context dependent activation of the AR and its transcriptome.

**Electronic supplementary material:**

The online version of this article (doi:10.1186/s12885-016-2453-4) contains supplementary material, which is available to authorized users.

## Background

Since the 1940s advanced prostate cancer has been treated with surgical or chemical castration in order to reduce systemic androgen levels [1]. The cumulative experience is that such androgen deprivation therapy (ADT) leads to efficient regression of invasive prostate cancer and to reduced levels of the serological marker prostate-specific antigen (PSA). Unfortunately, ADT seems not to increase long-term overall survival of prostate cancer [2], and castration-resistant prostate cancer (CRPC) in patients on ADT is typically diagnosed by rising serum PSA levels. Patients with CRPC have a poor prognosis [3], and patients with metastases have shown median overall survival of ≤19 months [4]. Androgens, in particular dihydrotestosterone, are activating ligands of the androgen receptor (AR) transcription factor. Novel highly potent drugs that block either androgen production or its stimulation of the AR have shown effect in CRPC and are associated with an extended median survival of several months [1, 5]. Nonetheless, CRPC remains incurable and progresses in spite of any current therapy. The AR has been shown to be critical to proliferation and survival of the bulk population of prostate cancer cells both in early prostate cancer and in CRPC, but different mechanisms are at play. In physiological prostate homeostasis the prostate epithelium is dependent upon a paracrine mechanism according to which androgen stimulates the stromal AR to induce expression of diffusible growth factors such as FGF7, FGF10, IGF1 and EGF which are essential for prostate basal epithelial cell proliferation [6]. Epithelial basal cell expression of the AR with androgen available leads to proliferation arrest and luminal terminal cell differentiation. During progression of prostate cancer the AR switches from an epithelial anti-proliferative transcription factor to an oncogene. This may occur in a stepwise fashion by still incompletely understood molecular mechanisms. Several possibly independent steps in CRPC cell generation encompass the loss of ligand-bound AR-dependent inhibition of proliferation, the oncogenic addiction to AR signaling and the replacement of paracrine AR signaling by autocrine growth factor signaling [7–9].

The molecular mechanisms that underlie *AR* transcriptional induction in normal prostate epithelial homeostasis and to which extent these mechanisms are retained in putative prostate cancer stem cells (CSCs) are not understood. One hypothesis that could explain that prostate cancer invariably escapes from ADT and androgen targeted therapy (ATT) would be the existence of a subpopulation of prostate CSCs that are AR negative and therefore insensitive to androgen deprivation. Evidence has been found to support the paradoxical possibility that ADT and ATT could lead to expansion of the pool of prostate CSCs [3] hypothetically due to loss of negative feedback by more differentiated cancer cells. Additional consequences of ADT and ATT could be to induce reprogramming plasticity of CSCs such as epithelial to mesenchymal transition (EMT) or neuroendocrine transdifferentiation [1, 5].

The understanding of essential molecular mechanisms of putative prostate CSCs is hampered by the low number of these cells in patient materials. If those cells are AR negative and AR non-responsive and give rise to AR positive and AR-dependent cells it is possible that some features of normal prostate cells are retained, although with loss of abilities to terminal differentiation and apoptosis induction. Better understanding of normal differentiation is likely to offer new insights into tumor initiation and may help explain the functional significance of common genetic alterations seen in prostate cancer [10]. Utilizing a previously published model of stepwise prostate carcinogenesis [11–15] and prostate cancer cell lines we therefore undertook a further examination of conditions for the restriction of AR and classical AR target gene expression in different cellular contexts.

## Methods

### Reagents, antibodies, cell culture and cell lines

Primary Prostate Epithelial Cells (PrECs; American Type Culture Collection (ATCC); Cat# ATCC-PCS-440-010) and prostate cancer cell lines LNCaP (ATCC-CRL-1740), VCaP (ATCC-CRL-2876) and 22Rv1 cells (ATCC-CRL-2505) were bought from LGC Standards GmbH (Wesel, Germany). The prostate cell lines EP156T, EPT1, EPT2 and PrECs were grown in MCDB153 medium (Biological Ind. Ltd., Israel) with 1 % for EP156T and PrECs, and 5 % fetal calf serum (FCS) for EPT1 and EPT2 cells, and supplemented with growth factors and antibiotics as described elsewhere [13, 15]. EPT3 cells were grown in Ham’s F12 medium (Lonza, Basel, Switzerland, Cat# 3 MB147) with 5 % FCS. Cells with exogenous AR were grown in equivalent medium but without androgens and with charcoal stripped FCS. LNCaP and 22Rv1 cells were grown in RPMI-1640 (Lonza, Cat# BW12-702 F) with 10 % FCS. VCaP were grown in DMEM (Lonza, Cat# BE12-604 F) with 10 % FCS. For experiments investigating the effect of high calcium, cells were grown in standard MCDB-153 medium supplemented with 1 % FCS, 1 % FCS and 600 μM Ca(NO_3_)_2_, 10 % FCS or grown in RPMI-1640 with 10 % FCS. To study epigenetic restriction cells were grown in standard medium with 10 μM 5-Aza-2′-deoxycytidine (5-Aza-dC) (Sigma Aldrich, St. Louis, MO, USA, Cat# A3656) for five days with addition of 250 nM trichostatin A (TSA) (Sigma Aldrich, Cat# T1952) the last two days. Medium was changed each day. DNA microsatellite validation of progeny identity of EP156T, EPT1, EPT2, EPT3-PT1 and EPT3-M1 cells has been published previously [15]. Matrigel-overlay cultures were performed with modifications based on Debnath J et al. [16] with a bed of growth factor reduced (GFR) Matrigel (Cat# 356231, BD Biosciences) and 2 % GFR Matrigel in the medium, medium was changed every 3–4 days. Cells were grown in a humidified atmosphere containing 5 % CO_2_ at 37 °C. Primary antibodies; AR (Cat# ab133273, ab9474), actin (Cat# ab8226), GAPDH (Cat# ab181602) and PSA (Cat# ab53774) were purchased from Abcam (Cambridge, UK).

### Vectors, transfection and transduction

The pLenti6.3/V5-DEST-AR expression clone was generated by LR recombination reaction between the entry clone pDONR-AR (Genecopoeia™, Rockville, MD, United States, Cat# GC-E2325), and the destination vector pLenti6.3/V5-DEST. Correct insertion of the AR gene was verified by sequencing with CMV forward primer and V5(C-term) reverse primer, according to the manufacturer’s protocol (Invitrogen, Life Technologies, Carlsbad, CA, United States, Cat# V533-06).

The pLenti6.3/V5-DEST-AR and ViraPower™ Packaging Mix were co-transfected in the 293FT producer cell line, according to the manufacturer’s protocol (Invitrogen, Cat# K370-20). EP156T and EPT3-PT1 cells were seeded in six-well plates and infected with the viral supernatant. After 48 h incubation the supernatant was removed and cells were maintained in androgen-free MCDB medium with 2 μg/ml blasticidine for the selection of stably transduced EP156T-AR and EPT3-PT1-AR cells. Negative control cells were made for each cell type using the pLenti6.3/V5-GW/*lac*Z control vector (Invitrogen, Cat# K370-20).

### Indirect immunofluorescence assay (IF) and Western blotting (Wb)

For IF, cells were grown on 12 mm glass coverslips (Assistent, Sondheim v. d. Rhön Germany, Cat. # 1014/12/1001) in 24 well plates, then washed with PBS, fixed (4 % fresh formaldehyde in PBS for 20 min. at room temperature), permeabilized (0.5 % Triton X-100 for 10 min.), blocked (100 mM glycin for 10 min) and stored (in PBS at 4 °C) with PBS washes between each step. Following blocking with 0.5 % BSA/PBS for 15 min. primary antibodies were added at room temperature for 1 hour at indicated dilutions in 0.5 % BSA/PBS. The FITC-labelled secondary anti-rabbit or mouse IgG (Southern Biotech, Cat# 4050–02, 1030–02) was added for 30 minutes at room temperature in 0.5 % BSA/PBS. Coverslips were mounted in Prolong Gold with DAPI (Molecular Probes, Life Technologies, Cat# P-36931) on glass slides and analyzed using Leica DM IRBE fluorescence microscopy.

For Wb analysis cells were lysed in RIPA-buffer with 1:100 Protease Inhibitor Cocktail Set I (Calbiochem, Cat# 535142). Protein concentrations were measured using the Pierce BCA Protein Assay Kit (ThermoFisher Scientific, Waltham, MA, Cat# 23225), and 5 μg protein lysates were separated by SDS electrophoresis in NuPAGE® 10 % Bis-Tris Gels (LifeTechnologies, Carlsbad, CA, United States, Cat# NP0303BOX) followed by blotting to PVDF membranes (GE Healthcare Life Sciences, Cat# RPN1416F) using Pierce 1-Step Transfer Buffer (ThermoFisher, USA, Cat# 84731) and Pierce G2 Fast Blotter (ThermoFisher). Membranes were blocked for one hour in PBS 0.1 % Tween and 5 % Skim milk powder (Sigma Aldrich, St. Louis, MO, USA, Cat# 70166). Primary antibodies were incubated for 1 hour in blocking buffer at RT, and HRP-labelled secondary antibodies (GE Healthcare, Little Chalfont, UK, Cat# NA931V, NA934V), were incubated as the primary antibodies 1/10000. Pierce ECL Western Blotting Substrate (ThermoFisher, Cat# 23106) or SuperSignal West Femto Maximum Sensitity Substrate (ThermoFisher, Cat# 34096) was used for detection with Chemidoc XRS using Quantity One 4.6.5 (Bio-Rad). Molecular weight marker used was MagicMark XP (Life Technologies, Cat# LC5602).

### PSA quantification assay

Cell culture supernatants were centrifuged in an Eppendorf centrifuge at 14 000 x g for 2 minutes at room temperature, and 0.5 ml of the supernatants were analyzed using the Elecsys total PSA immunoassay (#04641655 190) in a Cobas analyzer (Roche, Basel, Switzerland) according to the kit manual and according to the accredited routines of the Laboratory of Clinical Biochemistry (LKB) Haukeland University Hospital. The lower detection limit is 0.003 ng/ml total PSA. Values above 100 ng/ml are considered above the measuring range.

### RNA purification, TaqMan real-time RT-qPCR and Agilent microarrays

Total RNA was extracted using the miRNeasy kit from Qiagen (Qiagen, Venlo, Netherlands, Cat# 217004). The total RNA was DNase treated, ss-cDNA was synthesized and the RT-qPCR was run and analyzed as previously described [17], using pre-designed Taqman probes (Life Technologies) with the following Assay ID numbers: *ACTB* (Hs99999903_m1), *AR* (Hs00171172_m1), *KLK3* (Hs02576345_m1), *NKX3-1* (Hs00171834_m1), *TMPRSS2* (Hs00237175_m1). The Agilent Human Whole Genome (4x44 k) Oligo Microarray with Sure Print Technology (Agilent Technologies, Palo Alto, CA, US, Design # G4112-60520 G4845-60510), was used to analyze samples in the present study. Total RNA purification, cDNA labeling, hybridization and normalization have been described previously [17, 18]. Following normalization, significance analysis of microarray (SAM) of the J-Express program package (http://www.molmine.com) [19] was used for identification of differentially expressed genes. Only genes that changed at least 2.0 fold with FDR below 10 % were considered as differentially expressed genes in cell lines. ArrayExpress ID for the EP156T and EPT1 cells is (ID: E-TABM-949), EPT2 and EPT3 cells is (ID: E-MTAB-1521) [15] and for the EP156T, EP156T-LacZ, EP156T-AR, LNCaP, VCaP and 22Rv1 cell lines (ID: E-MTAB-3715).

### RNA sequencing (RNA-seq)

Total RNAs were included for RNA-seq if RIN (RNA Integrity Number) was above 9 and total RNA was at least 500 ng according to the Agilent 2100 Bioanalyzer™. Illumina HiSeq™ 2000 (Illumina) RNA-Seq was performed according to manufacturer’s instructions and according to StarSeq™ (Mainz, Germany) protocols. Prior to cDNA synthesis rRNA depletion of total RNA was done. The Qubit™/Bioanalyzer™ instruments were used for concentration and quality control and fragmentation and sizing was achieved using the Covaris^TMS2^ (Brighton, UK) kits and instrumentation according to instructions. cDNAs were tagged with barcoded adapters for multiplexing. Paired-end sequencing with read length 150 base pairs and 100 million reads per sample were chosen for raw sequence data acquisition. Raw data were formatted in BAM files and mapped to the December 2013 build of the UCSC Human genome browser. The following module versions were used in the TopHat and Cufflinks analyses for alignment and to estimate expression levels: TopHat2 v2.0.7, Bowtie 0.12.9, Cufflinks 2.1.1, Isaac Variant Caller 2.0.5, Picard tools 1.72. RNA-seq data is available at Gene Expression Omnibus (ID: GSE71797).

### Statistical analysis

Results from real-time RT-qPCR were analyzed using the RQ Manager v1.2 software and DataAssist v3.01 (both Applied Biosystems, Foster City, CA, USA). Error bars show 95 % confidence intervals. 95 % confidence intervals were analyzed for secreted PSA values using Microsoft Excel 2011 (Redmond, WA, USA).

## Results

### Restriction of AR and classical AR target gene expression in immortalized prostate basal epithelial cells

**Fig. 1:**
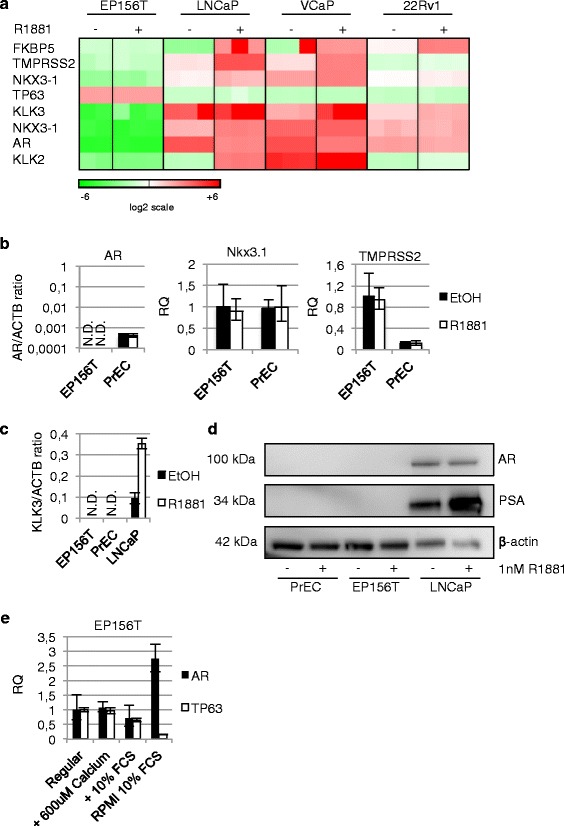
Expression data of EP156T and PrEC cells. **a** Agilent microarray gene expression data for the indicated gene symbols are shown in the heatmap according to supervised hierarchical cluster analysis (J-Express™ software) of different cell types with or without androgen R1881 in the growth medium. EP156T and LNCaP cells were treated with 10 nM for 48 hours and 22Rv1 and VCaP cells with 1 nM R1881 for 24 hours. Red color indicates high expression. **b** and **c** RT-qPCR comparing expression of *AR*, *NKX3-1*, *TMPRSS2* and *KLK3* between EP156T and PrECs. **d** Western Blot of AR and PSA in PrEC and EP156T cells compared to LNCaP cells with ± 1 nM R1881 stimulation for 48 hours. **e** RT-qPCR of AR and TP63 in EP156T cells after 6 days culture under different calcium and FCS concentrations. N.D. = not detected. Error bars show 95 % confidence intervals. RQ = relative quantity

The restricted expression of the androgen receptor and classical AR target genes were initially validated in prostate epithelial cells with basal cell features. The EP156T cells are hTERT immortalized prostate basal epithelial cells [11, 13, 18, 20] that can be passaged indefinitely as transit amplifying cells in subconfluent monolayer cultures. EP156T cells were examined at different passages with different concentrations of androgen in the growth medium. *AR* mRNA could not be detected in either of these conditions using Agilent oligonucleotide microarray analyses (Fig.1a), and this was supported by RNA-seq (Table 1) and validated by TaqMan reverse transcription quantitative PCR (RT-qPCR) assays (Fig. 1b). The transcription of a core set of classical AR target genes in prostate epithelial cells was focused on and consisted of *KLK3*, *TMPRSS2*, *KLK2*, *NKX3-1* and *FKBP5*. Of these, *KLK3* and *KLK2* mRNAs were non-detectable using highly sensitive assays (Fig. 1a/b/c and Table 1) and none of these target genes could be induced to higher expression following addition of the synthetic androgen R1881 at different concentrations to the growth media (Fig. 1a/b/c). As expected no AR protein was detectable in Western blots (Fig. 1d). In order to test the robustness of the repressed expression of the *AR* and AR target genes, numerous growth factors, combination of growth factors and growth conditions were tested as exemplified in Additional file 1: Table S1. FGF7 has been shown to promote luminal differentiation [21]. EGF is used in the MCDB medium, but has been shown to retard luminal differentiation, therefore removal of EGF and addition of the MAPKK inhibitor PD98059 was examined [22]. We also investigated if co-culture with mesenchymal EPT1 cells or if growth in a three-dimensional Matrigel-overlay culture could stimulate differentiation of EP156T cells. A highly sensitive PSA immunoassay was used to screen cell culture supernatants and this was negative at all conditions tested for the EP156T cells in contrast to the very high PSA values detected in growth medium of the LNCaP positive control cells (Additional file 1: Table S1).

**Table 1:** RNA-seq quantification of transcripts in cell lines with or without the androgen agonist R1881

GENES		R1881		R1881		R1881		R1881		R1881
	EP156T	EP156T	EPT3-M1	EPT3-M1	LNCaP	LNCaP	22Rv1	22Rv1	VCaP	VCaP
AR	0	0	3	3	64	36	31	26	125	44
KLK3	0	0	0	0	35	799	4	9	5	52
TMPRSS2	4	4	0	0	18	305	4	6	10	63
NKX3-1	0	0	4	4	40	139	34	40	171	319
KLK2	0	0	0	0	5	169	5	12	447	1010
FKBP5	15	13	40	39	5	331	30	254	7	181
TP63	45	40	0	0	0	0	0	0	0	0
MYC	37	35	21	20	34	8	38	37	91	19

EP156T, EPT3-M1 and LNCaP cells were treated with 10 nM R1881 for 48 hours and 22Rv1 and VCaP cells with 1 nM R1881 for 24 hours. Values are in fragments per kilobase of exon per million reads mapped (fpkm) and rounded to the nearest integer

### Expression of the *AR* and AR target genes in primary prostate cells and prostate cancer cell lines

Transcription of *AR* and AR target genes were then tested in parallel controls in primary epithelial prostate cells (PrECs) and the established prostate cancer cell lines LNCaP, VCaP and 22Rv1. LNCaP cells are widely used as an approximation to androgen sensitive cancer and 22Rv1 cells are considered one model of AR positive CRPC. PrECs reach senescence and die following a limited number of cell divisions. A low level of AR mRNA was detectable in PrECs according to sensitive RT-qPCR assays. But addition of androgen did not lead to increased expression of AR target genes as exemplified for the *KLK3*, *NKX3-1* and *TMPRSS2* mRNA (Fig. 1b/c). In Western blots no AR was detectable in PrECs (Fig. 1d). In contrast, striking AR target gene expression patterns were induced by androgen in the 3 cancer cell lines (Fig. 1a/d, Table 1). Addition of both 1 nM and 10 nM of the synthetic androgen R1881 led to decreased *AR* mRNA and protein in LNCaP cells in 48 hours as previously published [23, 24] (Table 1 and Fig. 1d). The RNA-seq data show that 1 nM R1881 for 24 hours decreased *AR* mRNA levels in VCaP cells 2.8 fold and 10 nM R1881 for 48 hours decreased *AR* mRNA levels in LNCaP cells 1.8 fold (Table 1). This androgen-repressive effect on *AR* mRNA was much less pronounced in the 22Rv1 cells. As shown in Table 1, androgen led to strong upregulation of the classical AR target genes in spite of reduced absolute levels of the *AR*, *e.g. KLK3* was upregulated 22.8 fold in LNCaP, 10.4 fold in VCaP and 2.3 fold in 22Rv1 cells (Table 1).

### Neither high calcium medium nor epigenetic modifiers are sufficient to induce *AR* expression

Notch signaling is required for normal prostate epithelial cell proliferation and differentiation [25]. EP156T cells are propagated in low calcium medium in which NOTCH1 signaling is constitutively activated while E-cadherin (CDH1) signaling is inhibited [26]. It has previously been published that changing to a high-calcium growth medium leads to differentiation of EP156T cells [27]. EP156T cells were grown in MCDB medium supplemented with 600 μM calcium or RPMI-1640 + 10 % FCS, also containing about 600 μM calcium. As *AR* expression levels in EP156T are around the detection limit of RT-qPCR, DNA input was increased 10-fold for *AR* assays. We observed that calcium supplementation of the regular MCDB growth medium resulted in negligible changes in expression of *AR* and *TP63* while growth in RPMI-1640 and 10 % FCS resulted in a 3-fold upregulation of AR mRNA and >80 % reduction of the basal marker *TP63*. Additionally, cells were grown in regular MCDB growth medium supplemented with 10 % FCS, resulting in an about 30 % decline in *AR* and *TP63* mRNA (Fig. 1e), suggesting that neither the calcium concentration nor the high FCS can account for the differentiating effect in contrast to what has previously been suggested [27]. To corroborate these findings, parallel experiments with PrECs showed a decrease of *TP63* expression in all conditions, while AR expression was upregulated about 1.5 fold by 600 μM calcium and 10 % FCS and no change seen in RPMI-1640 medium, adding further complexity to the role of extracellular calcium in prostate basal cell differentiation (Additional file 2: Figure S1a).

Genome-wide ChIP-chip data of EP156T and EPT1 cells have suggested epigenetically repressed patterns of DNA and histone lysine methylations in the promoter regions of the *AR* and classical AR target genes [12] (and results not shown). We therefore wanted to investigate if the restriction of *AR* transcription in basal epithelial cells is on an epigenetic level that can be reversed by using compounds that modify epigenetic markers. For this purpose we treated EP156T and PrEC cells with a combination of the demethylating agent 5-Aza-2′-deoxycytidine (5-Aza-dC) and the histone deacetylase inhibitor trichostatin A (TSA). We found that even if imprinted genes were robustly activated as assessed by RT-qPCR (Additional file 2: Figure S1b), *AR* was only marginally altered after 5 day treatment with 5-Aza-dC and addition of TSA at day 4 and 5 (Additional file 2: Figure S1b) and no androgen-dependent transcription of the classical AR target genes was detected.

### Epithelial to mesenchymal transition was associated with detectable increase of AR expression in EP156T cells

**Fig. 2:**
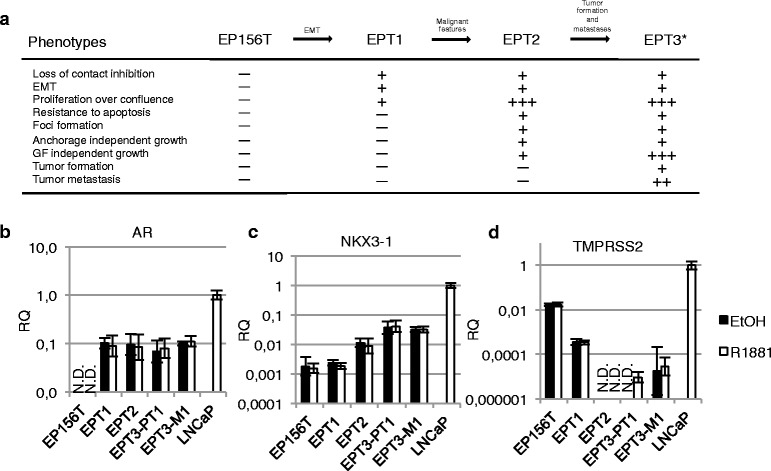
*AR* is expressed in a mesenchymal context, but target genes are repressed. **a** An experimental model of stepwise transformation of prostate cells to malignant cells. The model was started from benign EP156T epithelial cells obtained during surgery. The cells were grown to confluence and kept for almost 4 months without splitting to select for cells with reduced cell-to-cell contact inhibition. EPT1 cells appeared following EMT of EP156T cells. EPT1 cells were grown to confluence for several weeks and foci appeared in the monolayers. EPT2 cells were picked from the foci and selected and cloned by growth in soft agar. Neither EP156T nor EPT1 were able to grow in soft agar. Individual clones of EPT2 were next grown in protein free medium, and the selected cells were tumorigenic and generated EPT3 cells which were recovered from subcutaneous mice tumors and transduced with a GFP-luciferase vector [15].*Orthotopic injection of EPT3-GFP-luc cells in mice resulted in the EPT3-PT1 cells derived from the primary tumor. EPT3-M1 cells were isolated from abdominal metastasis. The accumulation of malignant features as one cell type was derived from its progenitor is listed. RT-qPCR of **b** *AR*, **c** *NKX*3-1 and **d** *TMPRSS2* expression in epithelial (EP156T) and derived mesenchymal cells compared to LNCaP, treated with 10 nM R1881. Error bars show 95 % confidence intervals. N.D. = not detected. RQ = relative quantity

When epithelial EP156T cells were selected in confluent monolayers for several months they gave rise to mesenchymal type EPT1 cells following EMT [13]. From the EPT1 cells a succession of mesenchymal type cells with accumulating malignant features were selected using different growth conditions (Fig. 2a) [15]. The genome-wide gene expression, epigenetic and functional changes of EP156T cells and the progeny mesenchymal type EPT1, EPT2 and EPT3 cells have been previously published using Agilent microarrays [11–13, 15]. This stepwise carcinogenic model was utilized to compare *AR* and AR target gene expression in epithelial and mesenchymal phenotypes with a common genotype. As shown in Fig. 2b, *AR* mRNA became detectable in EPT1 cells and remained at similar levels in the tumorigenic EPT3-PT1 and EPT3-M1 cells according to both Agilent microarray [15], and TaqMan RT-qPCR assays. The addition of 10 nM R1881 to the growth medium for 48 hours did not lead to any significant gene expression changes of either the AR or its classical targets. This was validated for the *AR* and the *NKX3-1* and *TMPRSS2* genes in all the mesenchymal type cells using TaqMan RT-qPCR (Fig. 2b/c/d). The EPT3-M1 cells which were derived from a metastasis of the orthotopic mouse tumor EPT3-PT1 were analyzed using RNA-seq technology (Table 1), revealing that neither the *AR* nor its classical target gene expression were affected by 10 nM R1881 for 48 hours. *KLK3* was not detectable in any of the mesenchymal type cells (Table 1 and results not shown). Even though *NKX3-1* and *FKBP5* mRNAs were detectable, their transcription levels were unaffected by the addition of androgen (Table 1). The endogenous expression of AR protein was detectable using indirect immunofluorescence (IF) assays with an anti-AR specific antibody as exemplified for EPT3-PT1 cells in Fig. 4b. The latter assay additionally showed that the endogenous AR was functional regarding cytoplasmic localization in androgen depleted conditions followed by nucleoplasmic accumulation when 1 nM R1881 was added to the growth medium. This low-level AR expression was, however, unable to direct androgen-dependent classical target gene expression in this mesenchymal context.

### Exogenous expression of the androgen receptor in EP156T and EPT3-PT1 cells

The initial series of experiments using a variety of growth factors, growth conditions and combinations revealed the robust restriction of *AR* expression in epithelial EP156T cells and the lack of androgen-dependent gene expression in PrECs. Even though the *AR* became detectable following EMT of EP156T cells, no androgen-dependent induction of AR target genes could be detected in the mesenchymal type cells. For this reason we constructed AR expression vectors in order to examine the hypotheses that AR expression above a threshold level would be required in order to activate the classical AR target genes in either the epithelial or the mesenchymal context.

**Fig. 3:**
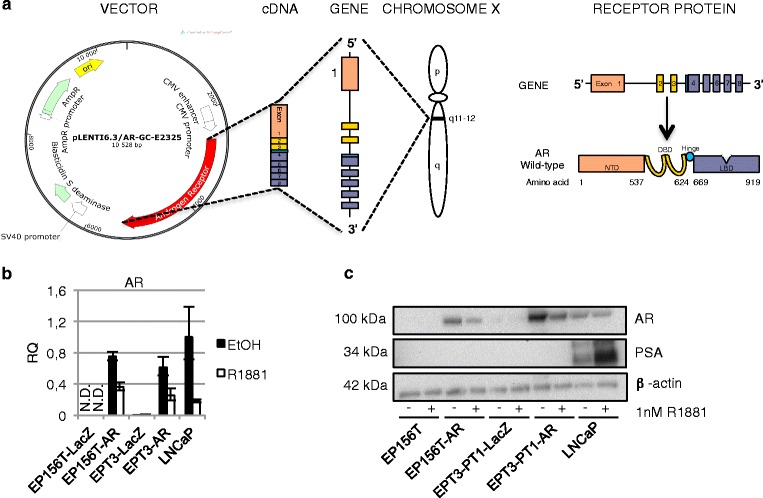
Exogenous expression of the Androgen Receptor. **a** The human androgen receptor (AR) is mapped to the proximal long arm of the X-chromosome (Xq11-12). The eight exons that encode the human AR protein are separated by introns of various lengths. Like other nuclear receptors, the AR protein consists of several functional domains such as the N-Terminal Domain (NTD), DNA-Binding Domain (DBD), the hinge region and the Ligand-Binding Domain (LBD). The pLENTI6.3/AR-GC-E2325 vector contains the human cytomegalovirus (CMV) immediate early promoter that allows for high-level, constitutive expression of the *AR* gene. The figure is adapted from [75]. **b** RT-qPCR and **c** Western Blot of AR in cells transduced with the AR in cultures ± 1 nM R1881 for 48 hours. Error bars show 95 % confidence intervals. RQ = relative quantity. N.D. = not detected

The lentiviral AR expression vector used in this study is shown schematically in Fig. 3a. Both the epithelial type EP156T and mesenchymal type EPT3 prostate cells were transduced to generate EP156T-AR and EPT3-PT1-AR cells, respectively. *AR* mRNA levels were comparable to expression levels of the androgen responsive LNCaP cell line according to TaqMan RT-qPCR assays (Fig. 3b). Western blots showed that AR expression levels of transduced EP156T-AR and EPT3-PT1-AR cells were comparable to endogenous AR expression in LNCaP cells (Fig. 3c).

### Functionality and androgen responsiveness of exogenous AR in the E and M contexts

**Fig. 4:**
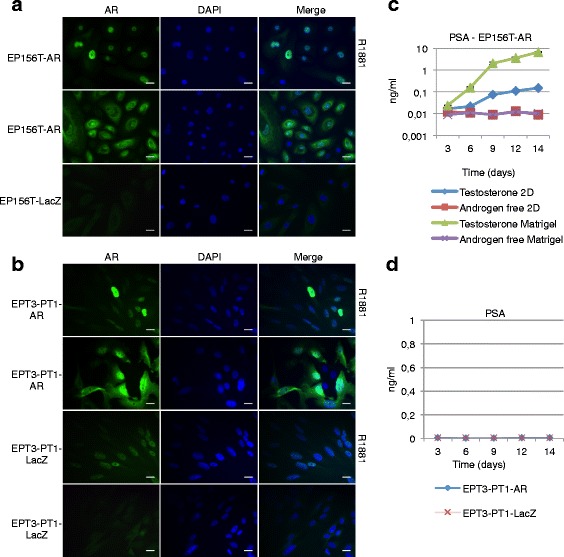
Exogenous Androgen Receptor is functional. **a** Exogenous AR in EP156T and **b** EPT3 translocates to the nucleus upon stimulation with 1 nM R1881. **c** PSA production in EP156T cells with exogenous AR in monolayer and matrigel-overlay method in regular medium containing 10 nM testosterone or androgen-free medium. **d** PSA production in EPT3-PT1-AR and -LacZ stimulated with 1nM R1881. Scale bars 20 μm. Error bars show ± 95 % confidence interval

In order to test the functionality of the exogenous AR protein, we first examined both EP156T-AR and EPT3-PT1-AR cells using indirect immunofluorescense of single cells with an anti-AR antibody. Figure 4a shows that the exogenous AR protein of EP156T-AR cells was localized mostly in the cytoplasm, but also in the nucleoplasm in androgen depleted medium. The established knowledge is that in the absence of androgen ligand the wild type AR is trapped in a cytoplasmic complex with HSP90 and other proteins. Upon androgen binding, the AR undergoes a conformational change and is released from the cytoplasmic complex, dimerizes and is imported into the nucleus [28]. Consistent with this, Fig. 4a shows that in EP156T-AR cells the addition of 1 nM R1881 to the medium is followed by a complete shift of AR into the nucleoplasm after 48 hours. In mock transduced EP156T-LacZ cells no AR was detectable either in the presence or in the absence of androgen (Fig. 4a). In the epithelial (E) context the androgen-dependent nuclear import of exogenous AR was therefore demonstrated. As can be seen in Fig. 4b, the endogenous AR is weakly detectable in the cytoplasm of the M type EPT3-PT1-AR cells and nuclear import is demonstrated following inclusion of 1 nM R1881 for 48 hours. Consistent with the Western blot quantitative results (Fig. 3c) a much stronger AR signal was found in the cytoplasm of EPT3-PT1-AR cells. Addition of 1 nM R1881 in the medium induced a complete shift to the nucleoplasm of both endogenous and exogenous AR after 48 hours (Fig. 4b).

### Exogenous AR directs functional PSA production in E, but not in M contexts

In order to test for functional PSA production monolayer cultures of epithelial EP156T-AR and mesenchymal EPT3-PT1-AR cells were grown with or without androgen. As shown in Fig. 4c, the androgen-dependent PSA concentration in the supernatant of EP156T-AR cells was detectable after 3 days following addition of androgen to sub-confluent monolayers of EP156T-AR cells. Increasing PSA production from the confluent monolayers was recorded in the following two weeks. In contrast, no PSA secretion was detected in EPT3-PT1-AR or control cells in the presence of androgen (Fig. 4d). The M type cultures were monitored for up to 14 days without evidence of PSA secretion.

### EP156T and EP156T-AR cells form spheroids in Matrigel, but only EP156T-AR cells secrete detectable PSA

**Fig. 5:**
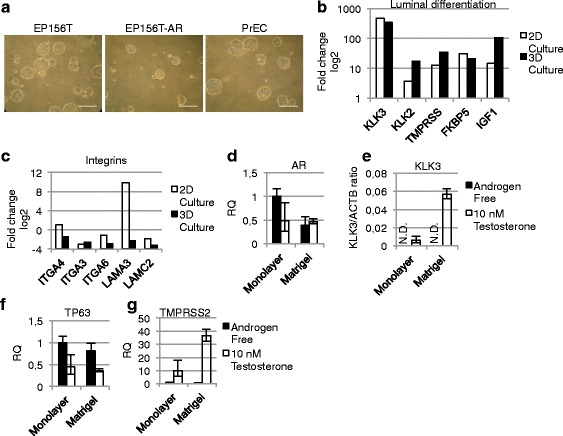
Forced Androgen Receptor expression induces target gene expression. **a** Phase-contrast of cells grown in Matrigel-overlay culture at day 12 with 10 nM testosterone. **b**-**c** Agilent microarray gene expression data were analyzed using SAM in the J-Express software to find fold change upregulation (positive numbers) or downregulation (negative numbers) of the shown genes in EP156T-AR cells stimulated by 10 nM Testosterone for 14 days compared to EP156T-AR cells grown in androgen free medium. **d**-**g** RT-qPCR of EP156T-AR cells in androgen free or medium with 10 nM testosterone in 2D or Matrigel-overlay culture. **d** *AR*, **e** *KLK3*/*ACTB* ratio, **f** *TP63* and **g** *TMPRSS2*. Error bars show 95 % confidence intervals. RQ = relative quantity. Scale bars 200 μm. N.D. = not detected

As exemplified in Fig. 5a, PrEC, EP156T and EP156T-AR cells formed glandular like spheroids in Matrigel while M type EPT cells did not exhibit this functional ability (results not shown). It was noted that EP156T-AR spheres were consistently smaller than spheres formed by EP156T and PrEC cells fitting with a proliferation suppressive effect of androgen-stimulated AR in basal epithelial cells (Fig. 5a). Similar to in monolayer cultures EP156T-AR cells were found to secrete PSA in an androgen-dependent way in Matrigel, but the amounts detected from the supernatants from the three-dimensional culture far exceeded that in monolayer (Fig. 4c). No PSA was detected using the highly sensitive PSA immunoassay to examine culture supernatant of EP156T cells in Matrigel. LNCaP cells secreted high amounts of PSA when grown in Matrigel (Additional file 1: Table S1). Total RNA was purified from androgen-stimulated cultures of both EP156T cells and PrEC cells. In PrEC the *AR* mRNA was detected in low amounts using real-time RT-qPCR, but *KLK3* mRNA was not detectable even with androgen available in the growth medium (results not shown).

### The androgen-dependent transcriptome of exogenous AR in two- and three-dimensional culture

In order to obtain a genome-wide perspective on androgen-dependent AR target genes in EP156T-AR cells, total RNA of cells that were grown either with or without androgen in monolayer cultures or grown in the presence of androgen in Matrigel cultures for 14 days were profiled using the Agilent 44 k microarrays. In monolayer culture 1836 genes were differentially regulated by a factor of at least 2 and a FDR < 10 in cells expressing exogenous AR, 924 genes were upregulated by androgens and 912 downregulated. In Matrigel culture 1673 genes were differentially regulated, 894 genes were upregulated by androgen and 779 downregulated. 855 genes were differentially expressed following androgen addition both in 2D- and 3D-culture.

As exemplified in Fig. 5, several categories of genes switched expression patterns in EP156T-AR cells in an androgen-dependent way, including classical AR target genes (Fig. 5b) and prostate characteristic integrins and laminins (Fig. 5c). Interestingly, the patterns of change of these genes were similar for androgen-induced EP156T-AR cells both in monolayer cultures and in Matrigel cultures. These transcription levels were also validated using RT-qPCR for *AR*, *KLK3*, *TP63* and *TMPRSS2* (Fig. 5d-g).

One advantage of the gene expression analysis is that the *AR* probe on the Agilent G4845 array targets the 3’-UTR (untranslated region) of the *AR* mRNA. This sequence is absent in the *AR* mRNA that is transcribed from the *AR* open reading frame of the expression vector. When the TaqMan real-time RT-qPCR assay is used to detect *AR* exon sequences in parallel, a distinction can be made between endogenous and exogenous *AR* mRNAs of the same cell cultures. It was of considerable interest to examine the possibility if basal AR expression might have a positive feedback effect on endogenous *AR* transcription. Expression levels of *AR* in the absence and presence of androgen were examined in cells with or without exogenous AR expression, but endogenous *AR* was not detectable. These experiments showed that in EP156T-AR cells the restriction of endogenous *AR* expression persisted even if the classical AR target genes were activated by exogenous AR and androgen.

## Discussion

AR negative (AR^**−**^) prostate epithelial stem cells divide asymmetrically to self-renew and to differentiate into either non-proliferating AR^**−**^ neuroendocrine cells or TP63^**+**^/AR^**−**^ transient amplifying (TA) cells in the normal adult prostate. The basally located AR^**−**^ TA cells undergo a limited number of amplifying rounds of proliferation before maturing into TP63^**+**^/PSCA^**+**^ intermediate cells [7, 29–31]. When AR expression is induced by incompletely understood mechanisms and with sufficient androgen available, intermediate cells terminally differentiate into luminal-secretory cells. An important aspect of the terminal differentiation is that androgen-bound AR represses MYC to inhibit proliferation and activates a large number of luminal secretory target genes [7, 9].

Many groups have investigated *in vitro* the molecular events associated with replication and differentiation of prostate basal cells to luminal secretory cells and found restricted AR and AR target gene expressions [26, 32–38]. One study has reported that co-treatment of prostate basal cells with clorgyline, 1,25-dihydroxyvitamin D_3_, all-trans retinoic acid and TGF-β1, induced expression of AR and loss of the basal marker KRT14 [39]. Lamb et al. found that confluent monolayers of primary prostate basal cells treated with dihydrotestosterone and FGF7 for 2–3 weeks differentiated into a top layer of luminal cells with expression of AR and classical AR target genes together with additional markers of terminally differentiated luminal secretory cells [21]. They found, however, that once cells reached passage 5, the efficiency of luminal differentiation was dramatically reduced. On only one occasion were they able to induce luminal differentiation in a patient-derived immortalized basal cell line. This is consistent with our results with the hTERT immortalized EP156T cells and the failure to demonstrate AR and classical AR target gene expression using either the conditions described by Lamb et al. or additional conditions including long-term confluent cultures, 3D Matrigel cultures, co-cultures with mesenchymal type cells and different combinations of biologically active compounds.

A few studies have reported morphological features of prostate basal cell differentiation using different treatments, such as the cell monolayer becoming stratified or forming gland like buds [40], but with either lack of AR expression or persistent expression of basal cell markers [35, 40–42]. Of interest, the original publication on the establishment of the EP156T cell line found that it formed glandular like structures in Matrigel and with indirect immunofluorescence detection of AR and KLK3 [20]. The EP156T cells, received at passage 37 in our laboratory and with a carefully kept passage history, still form glandular like structures in Matrigel, but using both highly sensitive real-time RT-qPCR assays and PSA detection assays we have been unable to detect AR and KLK3 production by these cultures. Additionally, treatment with the epigenetic modifying drugs 5-Aza-dC and TSA was not able to induce AR transcription, indicating that restriction of AR expression is not predominantly epigenetic but rather may be due to lack of cofactors.

In order to examine further the nature of the restriction of AR and AR target gene expression in EP156T cells, EP156T-AR cells stably expressing exogenous AR were selected. These cells were passaged in androgen depleted medium due to the potential of exogenous AR to induce terminal differentiation and growth arrest [7]. When androgen was added to EP156T-AR cells, both monolayer cultures and Matrigel cultures produced *KLK3* mRNA and protein. Several previous *in vitro* differentiation studies of prostate basal epithelial cells have noted a late restriction where *AR* and *KLK3* mRNAs can be detected without the corresponding proteins [37, 43–45]. The PSA assay verified that EP156T-AR cells secreted PSA to the supernatant in an androgen-dependent way. There was therefore no evidence of restricted translation in this model.

According to genome-wide microarray analyses, the addition of androgen to EP156T-AR cells induced the classical AR target genes both in monolayer and Matrigel cultures. This was in contrast to androgen treated EP156T cells or in EP156T-AR cells with androgen depleted medium. The endogenous *AR* mRNA remained repressed, however, both in monolayer cultures and in Matrigel. The possibility that androgen-bound AR could have a positive feedback effect on endogenous *AR* transcription was therefore not supported by the present studies. It remains a high priority future task to identify the precise molecular mechanisms of endogenous *AR* transcription activation in prostate basal epithelial cells. Possibilities include lack of essential cofactors, epigenetic repression or selection of mutants.

Downregulation of basal cell integrins α6β4 and α3β1 is considered a critical event in luminal differentiation [21], but it is unclear whether AR represses integrin mRNA transcription or whether loss of integrin expression must precede AR expression [30]. Interestingly, androgen addition to EP156T-AR cells was followed by downregulation of *ITGA3*, *ITGA4* and *ITGA6. LAMC2* was downregulated in an androgen-dependent way in EP156T-AR cells. LAMC2 is the laminin that binds integrins α6β4 and α3β1 in basal prostate cells. Several additional integrins and laminins changed their expression in androgen treated EP156T-AR cells, indicating that the AR is involved in co-ordinated changes of integrins and laminins in differentiating prostate basal cells.

The lineage hierarchy of prostate epithelial differentiation remains inadequately defined [46]. The origin and relationship between the benign prostate cells that initiate cancer and the cancer stem-like cells that propagate tumors are still vigorously investigated [10, 47, 48]. Recent reports suggest that luminal epithelial stem cells can act as the cell of origin of prostate cancer in the form of a castration-resistant Nkx3-1-expressing cell (CARN) [49]. Additionally both mouse and human epithelial luminal cells can establish prostate organoids *in vitro* [50, 51].

AR is central to growth and survival of both benign and malignant prostate epithelial cells, but the mechanisms seem to be very different in normal prostate homeostasis and cancer growth. In normal prostate epithelial cells the requirement for androgen is mediated through AR in the prostate stromal cells. Stromal androgen-bound AR induces secreted growth factors, so-called andromedins such as IGF-1, EGF, FGF7 and FGF10, to promote growth and survival of the epithelium [6]. During prostate carcinogenesis AR expression in the stroma decreases concurrently with increased AR expression in the tumor cells as prostate cancer progresses [52], and stromal cells surrounding metastatic prostate cells are AR negative, suggesting that cancer cells themselves start to supply the necessary andromedins, releasing themselves from the requirement of AR-positive stromal cells and androgens. Prostate carcinogenesis and progression therefore seem to involve acquisition of autocrine growth signals in addition to a switch of the AR from being a cell intrinsic inhibitor of proliferation to becoming a stimulator of proliferation [6, 8, 52].

The AR is critical for proliferation and survival of the bulk population of prostate cancer cells both at early stages and during CRPC as reflected by the effect of AR-inhibiting therapy [53–55]. But prostate cancer always escapes from these treatments in support of the hypothesis that a small sub-population of AR^**−**^ and androgen-independent prostate CSCs is the source of more differentiated AR^**+**^ bulk population of prostate cancer cells [56]. The hypothesis that ADT may lead to a “rebound” increase in the number of AR^**−**^ cells with basal cell and CSC features, is reviewed elsewhere (see [3] and references therein [57–61]). Additional studies have reported androgen-independent early human prostate adenocarcinoma cells and prostate CSCs with low AR [48, 62–67].

The existence of AR^**−**^ prostate CSC with basal cell features could help to explain the recurrence of transdifferentiated neuroendocrine cancers following highly potent ATT of CRPC [68, 69]. It is not clear, however, if either loss of negative feedback by differentiated prostate cancer cells on CSC proliferation or if therapeutic inhibition of the AR could contribute to increase the pool of prostate CSCs [3, 70, 71] or contribute to induction of EMT, epithelial mesenchymal plasticity and increased aggressiveness and reprogramming potential in prostate CSCs [5, 69, 72–74]. In this regard, it is noteworthy that mesenchymal type EPT3-AR cells, in contrast to epithelial type EP156T-AR cells, were androgen non-responsive. They were unable to produce detectable PSA in the culture supernatants even with higher levels of exogenous AR protein than in EP156T-AR and LNCaP cells for up to 2 weeks in androgen containing growth medium. The restricted PSA expression in the mesenchymal context suggests that if ADT increases the pool of mesenchymal type prostate cancer cells, then this might go undetected during PSA monitoring of disease progression.

## Conclusions

Androgen receptor expression and classical target gene expression were restricted and androgen non-responsive in PrECs and immortalized EP156T cells both in monolayer and Matrigel cultures. Expression of exogenous AR in EP156T-AR cells induced an extensive androgen-dependent trancriptome including classical target genes. No restriction of *KLK3* mRNA translation was observed and PSA was detected in confluent monolayers and in Matrigel cultures. However exogenous AR with or without androgen did not induce endogenous *AR* mRNA transcription. Low-level AR and high-level exogenous AR were unable to induce *KLK3* mRNA or other classical AR target genes in mesenchymal type prostate cells. In summary these results demonstrate the context dependent function of the AR, and that epigenetics and/or availability of cofactors greatly influence the AR transcriptome and ultimately if AR acts in a tumor suppressive or oncogenic manner. It also demonstrates that PSA might not be a good biomarker in cancers with high cellular plasticity, particularly for cancers that are induced towards a mesenchymal phenotype. However more knowledge is required to understand the specific conditions that govern AR-regulated phenotypes including its role in differentiation.

## Abbreviations

ADT, androgen deprivation therapy; ATT, highly active androgen targeted therapy; ChIP-chip, chromatin immunoprecipitation with DNA microarray chip; CRPC, castration - resistant prostate cancer; CSC, cancer stem cell; E, epithelial; EMT, epithelial to mesenchymal transition; FDR, false discovery rate; FPKM, fragments per kilobase of exon per million reads mapped; IF, indirect immunofluorescence; M, mesenchymal; PrECs, human primary prostate transit amplifying cells with basal cell features; RNA-seq, RNA-sequencing; RT-qPCR, reverse transcription quantitative polymerase chain reaction

## Additional files

Additional file 1: Table S1. Supernatant PSA assay. Various cell lines were stimulated with different combinations of growth factors and in several co-cultures. Represented here is a selection of these samples. Concentrations: R1881 (10 nm), FGF7 (10 ng/ml), PD98059 MAPKK inhibitor (30 mM), EGF (5 ng/ml) and co-culture with 50-75 % EPT1B8 cells. + denotes added growth factor. Mesenchymal EPT1 cells with p63KD (p63 knockdown), CDH1 or ΔNp63α are described in [14]. (XLS 46 kb) Additional file 2: Figure S1. Differentation in high calcium medium or using epigenetically modifying methods. **a** RT-qPCR of *AR* and *TP63* in PrEC cells after 6 days culture under differing calcium and FCS concentrations. **b** RT-qPCR of *XIST*, *AR* and *TP63* in EP156T and PrEC cells treated with 5’-Aza-dC for 5 days and TSA day 4 and 5. N.D. = not detected. Error bars show 95 % confidence intervals. RQ = relative quantity. (PDF 54 kb)
