# Supplementary figures and images for: Context dependent regulatory patterns of the androgen receptor and androgen receptor target genes
Source: BMC Cancer. 2016 Jul 4;16:377. doi: 10.1186/s12885-016-2453-4 (PMC4932678; doi:10.1186/s12885-016-2453-4)

**a**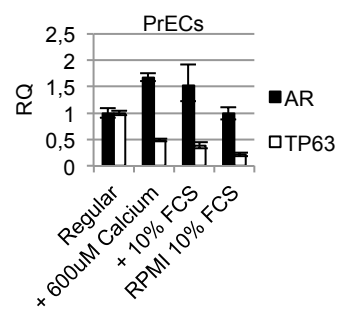**b**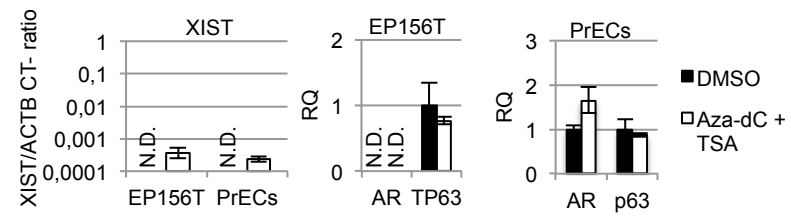

Supplement: Additional file 2: Figure S1. — Differentation in high calcium medium or using epigenetically modifying methods. a RT-qPCR of AR and TP63 in PrEC cells after 6 days culture under differing calcium and FCS concentrations. b RT-qPCR of XIST, AR and TP63 in EP156T and PrEC cells treated with 5’-Aza-dC for 5 days and TSA day 4 and 5. N.D. = not detected. Error bars show 95 % confidence intervals. RQ = relative quantity. (PDF 54 kb) [file 12885_2016_2453_MOESM2_ESM.pdf]
